# Tooth Clenching Induces Abnormal Cerebrovascular Responses in Migraineurs

**DOI:** 10.3389/fneur.2018.01112

**Published:** 2018-12-21

**Authors:** Nina Zaproudina, Antti-Pekka E. Rissanen, Jukka A. Lipponen, Anu Vierola, Saara M. Rissanen, Pasi A. Karjalainen, Seppo Soinila, Matti Närhi

**Affiliations:** ^1^Institute of Dentistry, University of Eastern Finland, Kuopio, Finland; ^2^Department of Clinical Physiology and Nuclear Medicine, Kuopio University Hospital, Kuopio, Finland; ^3^Institute of Biomedicine, University of Eastern Finland, Kuopio, Finland; ^4^Department of Sports and Exercise Medicine, Clinicum, University of Helsinki, Helsinki, Finland; ^5^Department of Applied Physics, University of Eastern Finland, Kuopio, Finland; ^6^Division of Clinical Neurosciences, General Neurology, Turku University Hospital and Department of Neurology, Turku University Hospital, Turku, Finland

**Keywords:** cerebral blood flow, headache, imaging, near-infrared spectroscopy, trigeminovascular system

## Abstract

Prevalence of masticatory parafunctions, such as tooth clenching and grinding, is higher among migraineurs than non-migraineurs, and masticatory dysfunctions may aggravate migraine. Migraine predisposes to cerebrovascular disturbances, possibly due to impaired autonomic vasoregulation, and sensitization of the trigeminovascular system. The relationships between clenching, migraine, and cerebral circulation are poorly understood. We used Near-Infrared Spectroscopy to investigate bilateral relative oxy- (%Δ[O_2_Hb]), deoxy- (%Δ[HHb]), and total (%Δ[tHb]) hemoglobin concentration changes in prefrontal cortex induced by maximal tooth clenching in twelve headache-free migraineurs and fourteen control subjects. From the start of the test, migraineurs showed a greater relative increase in right-side %Δ[HHb] than controls, who showed varying reactions, and right-side increase in %Δ[tHb] was also greater in migraineurs (*p* < 0.001 and *p* < 0.05, respectively, time-group interactions, Linear mixed models). With multivariate regression model, migraine predicted the magnitude of maximal blood pressure increases, associated in migraineurs with mood scores and an intensity of both headache and painful signs of temporomandibular disorders (pTMD). Although changes in circulatory parameters predicted maximal NIRS responses, the between-group differences in the right-side NIRS findings remained significant after adjusting them for systolic blood pressure and heart rate. A family history of migraine, reported by all migraineurs and four controls, also predicted maximal increases in both %Δ[HHb] and %Δ[tHb]. Presence of pTMD, revealed in clinical oral examination in eight migraineurs and eight controls, was related to maximal %Δ[HHb] increase only in controls. To conclude, the greater prefrontal right-side increases in cerebral %Δ[HHb] and %Δ[tHb] may reflect disturbance of the tooth clenching-related cerebral (de)oxygenation based on impaired reactivity and abnormal microcirculation processes in migraineurs. This finding may have an impact in migraine pathophysiology and help to explain the deleterious effect of masticatory dysfunctions in migraine patients. However, the role of tooth clenching as a migraine trigger calls for further studies.

## Introduction

Migraine is a common disabling neurological disorder with the prevalence of 18.2 % in females and 6.5 % in males (1). Abnormal activation of the trigeminovascular system as a result of disturbed brain stem function is a commonly accepted pathophysiological mechanism of migraine attacks; however, the primary triggering mechanisms are still poorly understood (2–4). Interictal cortical hyperexcitability, related to the attack frequency, has been demonstrated in migraine patients (5), and evidence for sensitization of the trigeminovascular pathway, involving both peripheral and central neurons, has been reported (6).

Masticatory apparatus is a pain-sensitive structure innervated by the trigeminal nerve. Pericranial muscles and tendon insertions in the orofacial area have been considered as potential sources of migraine-triggering stimuli (3). Masticatory parafunctions, such as tooth clenching and grinding, have been found to be more prevalent in migraineurs compared to non-migraineurs (7) and chronic tooth clenching has been suggested to predispose to migraine (8) although the mechanisms of the relationship, as well as the pathophysiology of masticatory parafunctions in general, are still unclear.

Dysfunction of autonomic nervous system, described in migraineurs (9), involves the autonomic regulation of the cerebral circulation (10). In migraine, Thomsen et al. (11) proposed dysfunction of the parasympathetic nervous system, playing a role in tonic vasodilation of the cerebral vessels, confirmed recently by others (12). Furthermore, previous studies on migraineurs have reported interictal vasoconstriction of cerebral microvasculature (13) and a decreased metabolic activity in several brain areas (14). Notably, Peroutka (9) proposed sympathetic dysfunction in migraine to be related with imbalance of sympathetic co-transmitters, specifically low level of norepinephrine, which normally counteracts the effects of the trigeminal system.

Near-Infrared Spectroscopy (NIRS), evaluating a regional balance between microvascular O_2_ delivery and utilization, has been applied to monitor cerebral hemodynamics (15). Using NIRS, a reduction of cortical oxyhaemoglobin ([O_2_Hb]) after sumatriptan injection has been reported during a migraine attack (16). Also, NIRS measurements performed interictally on migraineurs have demonstrated abnormal cerebrovascular responses e.g., to hypercapnia (17). In healthy individuals, NIRS measurements showed both primary motor and sensory cortical activation with [O_2_Hb] increase during voluntary tooth clenching associated with the task intensity (18). In addition, functional magnetic resonance imaging has shown that tooth clenching induces more complex and extensive cerebral activity changes than a hand motor task (19). To our knowledge, cerebral oxygenation responses to tooth clenching have not been studied in migraineurs.

In the present study, we hypothesized that divergences in cerebrovascular responses to tooth clenching in migraineurs may elucidate the role of tooth clenching as a headache trigger and aggravating factor for migraine. For this purpose, bilateral changes in local (de)oxygenation and blood volume in prefrontal cerebral cortex induced by maximal tooth clenching (MTC) were measured with NIRS in migraineurs and controls.

## Materials and Methods

This study was carried out in accordance with the recommendations of Research Ethics Committee of the Hospital District of Northern Savo. The protocol was approved by the Research Ethics Committee of the Hospital District of Northern Savo, Finland. All subjects gave written informed consent in accordance with the Declaration of Helsinki.

The study groups consisted of twelve subjects (ten women) suffering from migraine, diagnosed by a physician, and 14 control subjects (ten women). The main characteristics of the study subjects are presented in Table 1. Patients fulfilled the diagnostic criteria of migraine according to the International Classification of Headache Disorders (20) and were examined during a headache-free period. The headache was more clearly restricted to the right side in six, to the left side in three and was either bilateral or varyingly unilateral in three of the migraineurs. Seven patients had migraine with aura and five used triptans to treat attacks. The subjects did not have any other neurologic or cardiovascular diseases, nor any prophylactic treatment.

**Table 1:** Characteristics of the study subjects; mean (SD).

**Variable**	**Migraineurs (*n* = 12)**	**Controls (*n* = 14)**
Sex (M/F)	2/10	4/10
Age (years)	37.8 (11.3)	38.6 (10.0)
Height (cm)	172.0 (11.0)	169.3 (10.2)
Weight (kg)	71.1 (15.6)	68.0 (11.9)
Family history of migraine	12/12^**^	4/14^**^
Mood scores (0–21)	2.3 (2.8)	0.7 (1.2)
Duration of maximal tooth clenching (s)	80.0 (18.6)	83.2 (33.3)
Painful signs of TMD	8/12	8/14
Duration of migraine (years)	20.9 (10.0)	
Annual attacks frequency	18.4 (18.3)	
Systolic blood pressure at baseline (mm Hg)	158.3 (20.8)	157.5 (16.5)
Systolic blood pressure, maximal increase (mm Hg)	30.4 (11.0)^*^	19.6 (7.1)^*^
Diastolic blood pressure at baseline (mm Hg)	85.1 (15.3)	89.9 (14.2)
Diastolic blood pressure maximal increase (mm Hg)	18.0 (5.0)^*^	12.8 (4.3)^*^
Heart rate at baseline (beats/min)	71.7 (11.6)	66.7 (10.1)
Heart rate, maximal increase (beats/min)	9.4 (5.5)	12.5 (6.4)

*For group comparisons, either Mann-Whitney U- or t-test or Fisher's exact test were used. pTMD, painful signs of TMD, temporomandibular disorders*.

* *p < 0.05*,

** *p < 0.005 between the groups*.

In all participants, clinical oral examinations were carried out by one specialist in temporomandibular disorders (TMD) using the Diagnostic Research Criteria for TMD (21). Pain in masticatory muscles or temporomandibular joints during palpation or jaw movements was recorded (painful signs of TMD, pTMD) and its intensity evaluated by scale 0–3. Only two migraineurs and two control subjects reported history of TMD but the clinical oral examination revealed pTMD in eight and non-painful signs of joint-related TMD in three migraineurs. Pain in masticatory muscles was found also in eight controls. The subjects did not, however, report significant pain during MTC and duration of clenching did not differ between the groups or in relation to pTMD. Mood scores [0–21, (22)] and, in migraineurs, the intensity of headache (0–10) were self-reported.

The experiments were performed in an air-conditioned laboratory and were preceded by abstinence from eating and drinking for 2 h and significant physical activity for 1 d.

**Figure 1:**
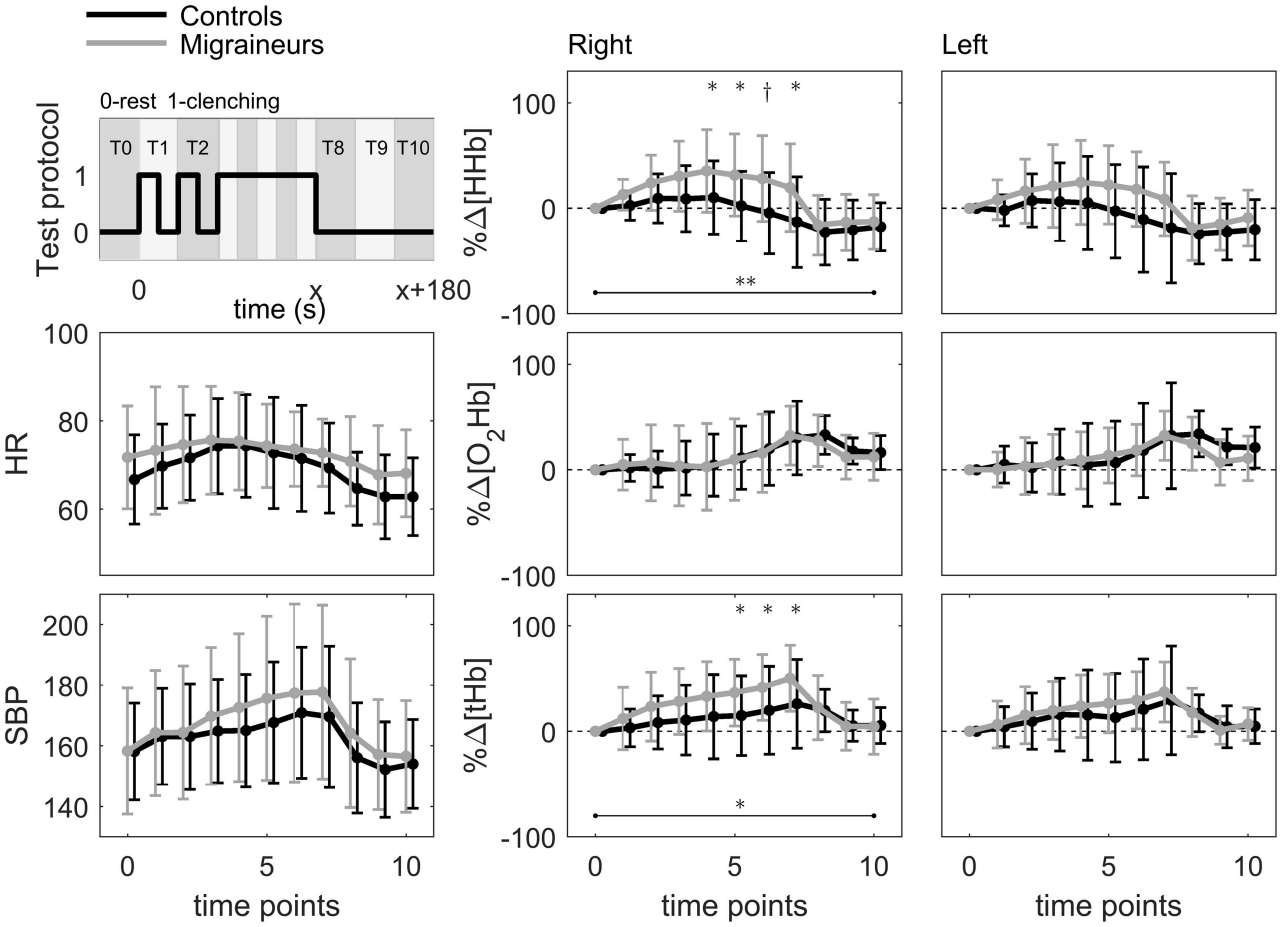
Test protocol and mean values of heart rate (HR), systolic blood pressure (SBP), and normalized relative concentration changes in deoxy- (%Δ[HHb]), oxy-(%Δ[O_2_Hb]), and total hemoglobin (%Δ[tHb]) on the right and left sides of prefrontal cerebral cortex of migraineurs (*n* = 12) and controls (*n* = 14) during maximal tooth clenching test (^*^p < 0.05, *p* < 0.005, ^**^p < 0.001 between the groups, Linear mixed models with Bonferroni corrections).

The protocol of MTC included two series of 5 s clenching + 5 s break, followed by one maximal clenching until volitional exhaustion [Figure 1, (23)]. During the maneuver, a continuous wave NIRS device (Oxymon MkIII Near-Infrared Spectrophotometer, Artinis Medical Systems, Zetten, The Netherlands) was used. NIRS probes consisted of one transmitting and one receiving optode and were placed over the frontal cortex on both sides, about 2 cm above the eyebrows. The interoptode distance was set to 35–40 mm so that a good signal quality was reached. The theory of NIRS and its use in physiological measurements have been described in detail elsewhere (15). Briefly, the intensity of incident and transmitted light is recorded continuously and, along with the specific optical pathlength and extinction coefficients, used for online estimation and display of concentration changes from the resting levels. Cerebral (de)oxygenation was estimated by recording relative concentration changes of oxy- (Δ[O_2_Hb]) and deoxyhemoglobin (Δ[HHb]), whereas local prefrontal blood volume was assessed by total hemoglobin (Δ[tHb] = Δ[O_2_Hb] + Δ[HHb]) (15). The differential pathlength factor (DPF) value used was calculated (DPF = 4.99 + 0.067 × Age^∧^0.814) according to the manufacturer's guidelines, and a sampling frequency of 10 Hz was used for collecting the data. The obtained data were averaged to give values in 1-s intervals. The obtained NIRS responses were normalized (%Δ[O_2_Hb], %Δ[HHb] and %Δ[tHb]), so that 100% represents a maximum-to-minimum amplitude of changes during MTC.

The systolic (SBP) and diastolic (DBP) blood pressures were measured from the 2nd and 3rd left fingertips using a Portapres device (Finapres Medical Systems, Amsterdam, Netherlands). Electrocardiogram was recorded using a modified chest lead 5 (V5).

Muscular activity from both masseter muscles was controlled with surface electromyography with ME6000 biosignal monitor (Mega Electronics Ltd; Kuopio, Finland).

For SBP, DBP, heart rate (HR), and NIRS data, the average values were calculated for ten time points: two points with 10-s intervals, then during maximal clenching (20, 40, 60, 80, and 100 % of clenching time), and first, second and third minutes after clenching (Figure 1), maximal and minimal values were also defined.

The SPSS Statistics 21.0 (IBM Corp., Armonk, NY, USA) was used for the statistical analyses. Changes in blood pressure, HR and normalized NIRS data were compared between the study groups with a Linear mixed model, adjusted for multiple comparisons (Bonferroni). A stepwise linear regression analysis was used to identify the predictors of the measured changes. Fisher's exact test, *t*-test and Mann-Whitney U-test were also used. The level *p* < 0.05 was considered significant.

## Results

The study groups did not differ regarding age, anthropometric characteristics or blood pressure and HR levels before the test, but migraineurs had more often family history of migraine than controls (*p* < 0.005, Table 1). MTC induced changes in averaged SBP, DBP and HR values in both groups (time effect, *p* < 0.001 for all) but there were no time-group interactions in the dynamics of blood pressure or HR (Figure 1). However, in migraineurs, as compared to controls, more prominent %Δ[HHb] increase occurred from the start of clenching, better seen on the right side (Figure 1). In addition, migraineurs showed a greater right-side relative increase in %Δ[tHb].

We entered explanatory variables (age, sex, migraine, and the family history of migraine, pTMD and its intensity, mood scores and duration of MTC, and for NIRS parameters, also SBP, DBP and HR) one at a time into the model to check their associations with the measured changes. There were no relationships between the blood pressure or HR and explanatory variables, but %Δ[HHb] and %Δ[tHb] values were associated with SBP, DBP, and HR (*p* < 0.001 for all). In addition, %Δ[HHb] values were associated with sex (*p* < 0.05, Linear mixed model). The greater values of %Δ[HHb] were related to the right side of measurement (*p* < 0.05) and, in migraineurs, there was such a tendency for %Δ[tHb] (*p* < 0.1 after Bonferroni correction). However, there were no time-group interactions in the dynamics of side-to-side differences in NIRS findings.

The dynamics of right-side NIRS changes differed between the groups for both %Δ[HHb] (adjusted for SBP, HR and sex, time-group interaction, *p* < 0.001, with significant differences at 40–100 % of MTC, maximal at 80% of MTC: Est. −43.7; CI −67.0 to −20.3, *p* < 0.005) and %Δ[tHb] (adjusted for SBP and HR, *p* < 0.05, with significant differences at 60–100 % of MTC, maximal at 100 % of MTC: Est. −33.4; CI −54.5 to −12.3, *p* < 0.05, Linear mixed models with Bonferroni corrections, Figure 1). There were no significant differences in the left-side NIRS changes between the groups.

**Figure 2:**
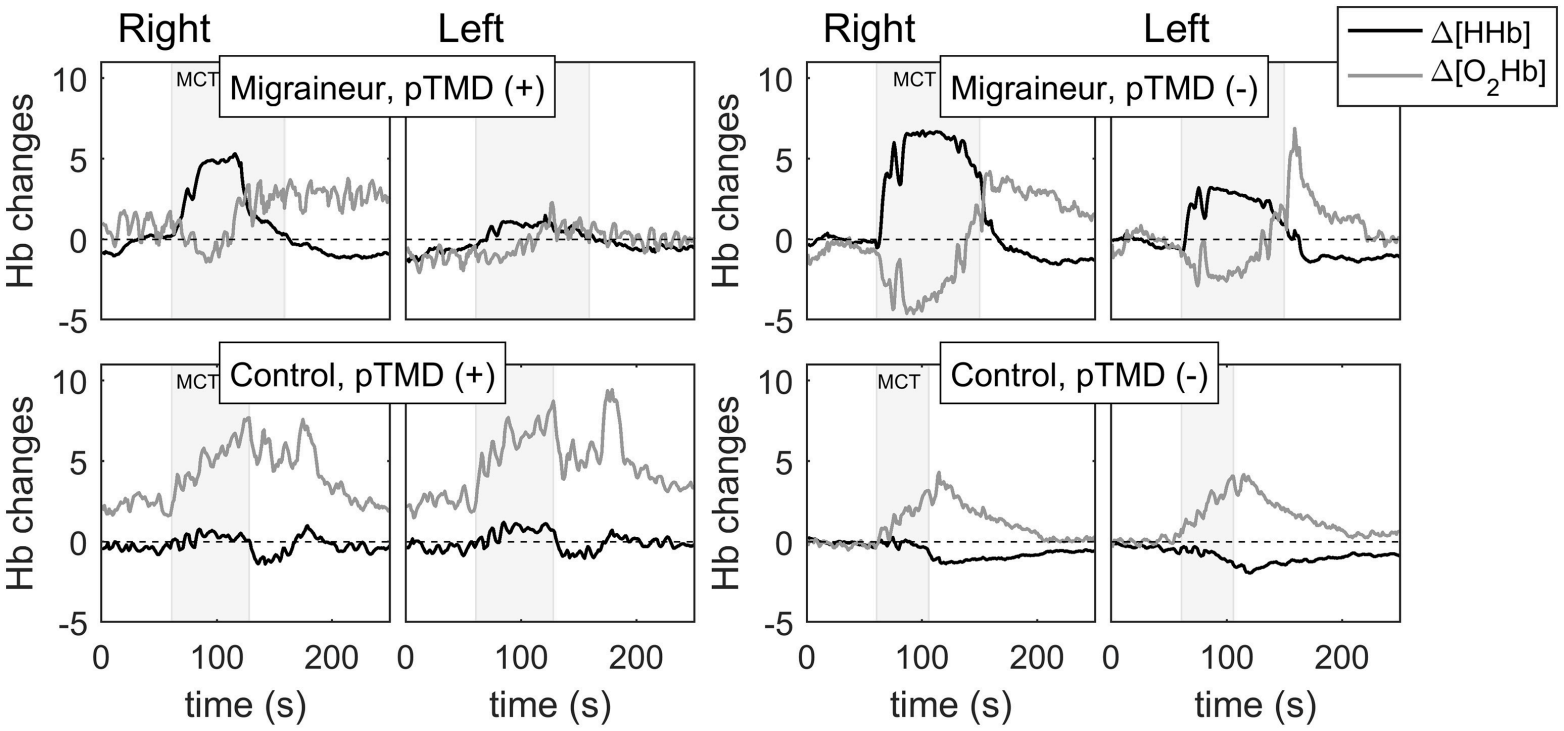
The tooth clenching-induced changes in concentrations of oxy-(Δ[O_2_Hb]) and deoxyhaemoglobin (Δ[HHb]) on the right and left sides of prefrontal cerebral cortex of a migraineur with [pTMD (+)] and one without painful TMD signs [pTMD (–)], a control subject with [pTMD (+)] and a healthy control.

Right-side %Δ[HHb] increase occurred in all migraineurs, however, with varying magnitude and in some of them, small %Δ[HHb] increase was followed by decrease. Controls showed varying reactions, %Δ[HHb] decreased in four, fluctuated in six and increased in four subjects. MTC induced also an initial right-side relative decrease of %Δ[O_2_Hb] in nine migraineurs and six controls (four of them with pTMD), but there were no significant differences in dynamics of %Δ[O_2_Hb] between the groups. The most representative curves of the relative changes in oxygenation parameters are presented in Figure 2.

The maximal registered changes were also analyzed. Maximal increases in SBP and DBP, compared to baseline levels, were greater in migraineurs than in controls (*p* < 0.05 for both, Mann-Whitney U-test, Table 1) whereas the maximal changes in NIRS parameters did not differ between the groups. To identify the modifiers of the maximal MTC-induced circulatory and cerebrovascular changes, all possible determinants (all variables listed above and, in migraineurs, also duration of migraine, frequency of attacks, aura, side and intensity of headache, and use of triptans) were entered stepwise into the Linear regression model. Migraine was found to predict the magnitude of maximal blood pressure increase, and, in migraineurs only, changes in circulatory parameters were associated with mood scores and the intensity of both headache and pTMD (Table 2). In controls, maximal %Δ[HHb] increase was related to presence of pTMD whereas an increase in cerebral oxygenation was inversely related to duration of MTC and pTMD intensity (*p* < 0.05 for all, Table 2). The baseline levels and changes in HR and blood pressure, added into the model, appeared to determine maximal changes in NIRS parameters. In addition, a family history of migraine, reported by all migraineurs and four controls, appeared to predict maximal changes in %Δ[HHb] and %Δ[tHb]. In migraineurs, on the left side, aura, pTMD intensity and use of triptans seem to play a role (Table 2).

**Table 2:** Predictors of tooth clenching-induced maximal changes in heart rate (HR), systolic (SBP) and diastolic (DBP) blood pressure, and normalized relative concentration changes in deoxy- (%Δ[HHb]), oxy-(%Δ[O_2_Hb]) and total hemoglobin (%Δ[tHb]) on the right and left sides of prefrontal cerebral cortex in all studied subjects (*n* = 26) and separately in controls (*n* = 14), and migraineurs (*n* = 12) with the clinical characteristics of migraine added into the model.

**Parameter**	**All subjects, predictor, β*;* Sign**.	**Controls predictor, β; Sign**.	**Migraineurs predictor, β; Sign**.

**HR AND BLOOD PRESSURE, MAXIMAL INCREASE**			
HR	Baseline levels, −0.53^**^	ns	Mood scores, 0.65^*^
SBP	Migraine, 0.56^**^	ns	ns
DBP	Migraine, 0.58^***^	ns	Headache (VAS), 0.64^***^
			pTMD intensity, 0.49^***^
			Sex, 0.35^**^
**HR AND BLOOD PRESSURE, MAXIMAL DECREASE**			
HR	Baseline levels, −0.48^*^	ns	MTC duration, −0.68^*^
**NIRS PARAMETERS, MAXIMAL INCREASE (WITHOUT HR AND SBP IN THE MODEL):**			
**Right side**			
%Δ[HHb]	ns	pTMD, 0.64^*^	ns
%Δ[O_2_Hb]	MTC duration, −0.41^*^	MTC duration, −0.62^*^	ns
		pTMD intensity, −0.52^*^	
**Left side**			
%Δ[tHb]	FHM, 0.49^*^	ns	Aura, −0.73^**^
**NIRS PARAMETERS, MAXIMAL INCREASE (WITH HR AND SBP IN THE MODEL)**			
**Right side**			
%Δ[HHb]	FHM, 0.50^**^	pTMD, 0.55^*^	HR increase, 0.77^**^
	Sex, −0.37^*^	SBP increase, 0.48^*^	
%Δ[O_2_Hb]	ns	MTC duration, −0.70^***^	ns
		HR decrease, 0.54^***^	
		pTMD intensity, −0.53^***^	
		SBP decrease, 0.28^*^	
%Δ[tHb]	HR increase, 0.63^***^	HR increase, 0.63^*^	SBP at baseline, 0.89^***^
	FHM, 0.45^*^		
**Left side**			
%Δ[HHb]	ns	SBP increase, 0.83^***^	ns
		MTC duration, 0.43^*^	
%Δ[O_2_Hb]	HR decrease, 0.64^***^	HR at baseline, −0.78^***^	ns
	SBP increase, 0.38^*^		
%Δ[tHb]	FHM, 0.46^*^	HR at baseline, −0.68^*^	Aura, −1.39^***^
			pTMD intensity, −0.9^***^
			Use of triptans, 0.35^*^

*Data were analyzed by logistic multivariate stepwise regression model (age, sex, baseline levels and changes of heart rate (HR) and systolic blood pressure (SBP), migraine and the family history of migraine (FHM), painful signs of temporomandibular disorders (pTMD), and its intensity (scale 0–3), mood scores, duration of maximal tooth clenching (MTC), and, in migraineurs, also duration of migraine, annual frequency of attacks, aura, use of triptans, side and intensity (Visual Analog Scale 0–10) of headache as explanatory variables)*.

* *p < 0.05*,

** *p < 0.01*,

*** *p < 0.005*.

## Discussion

In the present study, migraineurs showed different cerebrovascular responses to MTC in the right prefrontal cortex as compared to non-migraineurs. We believe this is the first report on such a finding. The observed differences in MTC-induced right-side cerebrovascular responses between migraineurs and non-migraineurs reflect differences in local microvascular O_2_ delivery and utilization between the groups.

We observed elevated %Δ[HHb] on the right side in response to MTC in migraineurs. Murata et al. (24) have previously observed motor-task-induced increase in [HHb] in patients with impaired cerebral blood flow (CBF) and hypothesized that, during neuronal activation in such a condition, [HHb] might increase due to lactate oxidation. Cerebral vasoconstriction in migraineurs has also been described (13) and an association between the endothelial dysfunction and migraine supports an enhanced role of migraine-related vasculopathy especially in migraine with aura (25). Accordingly, we hypothesize that the time course of the tracked changes in %Δ[HHb] in the migraineurs in our study likely depends on absolute baseline CBF: When less blood is available (i.e., prefrontal microvascular O_2_ delivery is relatively low reflecting prefrontal vasoconstriction), neural activation may lead to more rapid increase in local fractional O_2_ extraction resulting in increased %Δ[HHb]. Similar dynamics of [HHb] has been shown in patients with occlusal dysesthesia and suggested similarly to indicate reduced blood flow to the frontal pole cortex (26).

In addition to or instead of possible cerebral vasoconstriction in the migraineurs in this study, migraineurs' exaggerated local microvascular O_2_ utilization during MTC may also explain the findings of the elevated %Δ[HHb] on the right side. In the present study, %Δ[HHb] increase was seen in migraineurs but also in a few controls, most of them with pTMD. Thus, the more prominent increase in %Δ[HHb] may reflect a higher neuronal activation and hence O_2_ utilization as an exaggerated response to aversive stimulation, similar to those shown in cortico-limbic areas in response to negative emotional stimuli (27). It is, however, feasible to suggest that both migraineurs and controls with pTMD may belong to the same cluster of individuals having similar biopsychosocial risk factors for the development of pain conditions as has been presented for TMD in general (28). Notably, the maximal increases in both %Δ[HHb] and %Δ[tHb] were associated with family history of migraine and, thus, with a specific hereditary type of reactivity. The abnormal cerebrovascular responses observed in migraineurs are thus consequences of cerebral vasoconstriction and/or enhanced neural activation due to sensitization of the pain-controlling structures (5, 6). Moreover, cortical excitability factors and endothelial dysfunction have been proposed to interact in migraineurs (25).

In addition to %Δ[HHb], also %Δ[tHb] increased more in migraineurs than in non-migraineurs during MTC. Unfortunately, both %Δ[HHb] and %Δ[tHb] are relative parameters by their nature; thus, we do not know if the resting level of prefrontal microvascular tone was low or high before the MTC experiment. However, the exaggerated MTC-induced increase in %Δ[tHb] in migraineurs was mainly driven by the increase in %Δ[HHb], which simply reflects MTC-induced exaggeration of local imbalance between prefrontal O_2_ delivery and utilization in migraineurs. Accordingly, there were no significant differences in the responses of %Δ[O_2_Hb], which is another component of %Δ[tHb], to MTC between migraineurs and non-migraineurs. Still, initial %Δ[O_2_Hb] decrease was evident in some of migraineurs and controls; recently, it has been shown that experimental hypoxia may trigger migraine and aura (29). Overall, our NIRS findings may reflect interactions between migraine and masticatory disorders (30).

Notably, more prominent NIRS changes were found on the right side. Changes in CBF of the right prefrontal cortex, induced by psychological stress and measured by functional magnetic resonance imaging, have been related to stress levels, with a sustained activation after the task in subjects with a high stress level (31). Wang and coworkers have also demonstrated correlations between the baseline CBF and changes in HR and cortisol levels (31). Using NIRS, Tanida et al. (32) have found a correlation between the right-side prefrontal activity and HR increase, too. Migraineurs are more sensitive to stress than healthy subjects (33) and, in the present study, NIRS findings were associated with HR and SBP changes, dependent in migraineurs on the mood scores and an intensity of both headache and pTMD.

The relationship between blood pressure and NIRS responses has been shown earlier (34), which is physiologically expected considering the obvious effects that arterial blood pressure has on CBF and cerebrovascular resistance (35). Although it is thus acknowledged that greater blood pressure responses may have affected the NIRS parameters also in our current study, it is important to note that the between-group differences in the right-side NIRS findings remained significant after adjusting them for SBP as well as HR. Furthermore, the findings of the abovementioned studies (31–33) support the hypothesis that exaggerated stress reactivity is at least partly responsible for the coincidence of higher SBP increase and divergent cerebrovascular responses in migraineurs. In lambs, cerebral sympathetic nerve activity increases with imposed elevations of arterial pressure (36); the authors suggested that exceeding the upper limit of the autoregulation curve may trigger a reflex increase in cerebral sympathetic vasoconstrictor activity, playing a protective role in the CBF. However, an impairment of cerebral autoregulation was suggested in migraineurs (17) and, thus, an increase in blood pressure may occur in the circumstances of a failure of normal homeostatic mechanisms for autoregulation of CBF (25). Alternatively, it may partly explain the nature of cerebral vasoconstriction in migraineurs. However, despite the exact mechanisms behind our findings are unclear, the migraine-related MTC-induced changes in cerebral circulation may be mechanistically important but must be verified with brain imaging techniques with the possibility for evaluation of the absolute levels of CBF.

A question is which stimuli trigger headache attacks in migraineurs. A recent meta-analysis (4) evaluated studies from 1958 to 2015 with 27,000 primary headache patients showing 420 separate external triggers. Recently, generalized hypersensitivity to negative stimuli was described in migraine, focusing on both the sensory and emotional components (27). Based on the present study, we propose that increased reactivity to trigeminal stimuli together with dysregulation of CBF may be coupled in migraine pathophysiology: Augmented trigeminal reflex, described previously in migraineurs (23), may play a role in our finding of greater relative increase in prefrontal blood volume (i.e., %Δ[tHb] increase) in migraineurs. In fact, abnormalities in brain metabolic activity have been reversed after external trigeminal nerve stimulation and the change correlated with clinical improvement of migraine (14). Still, the functional relationship between the sympathetic nervous and trigeminovascular systems is under-appreciated (9). However, localization of the stimuli in trigeminal territory may be especially significant for migraineurs. In rats, trigeminal nociceptive stimulation has been shown to increase susceptibility to cortical spreading depression (37). Although corresponding human data are not available, in migraineurs, the thresholds to trigeminal stimulation were found to be lowered (38). Previously, Dawson (39) used tooth clenching as a model of muscular pain and showed higher serotonin levels and lower masseter muscle blood flow in patients with myofascial TMD as compared to controls. While our subjects did not report pain during MTC, clenching-related stimulation led to the above-mentioned differences in NIRS responses between the groups, while pTMD seemed to be more strongly associated with the CBF changes in controls than in migraineurs. The mechanisms of causal relationships between migraine and painful masticatory muscles are unknown. However, in tension-type headache, such an increased pain sensitivity has been suggested to be rather a consequence of frequent headaches than a risk factor for them (40). In our study, despite pTMD was revealed also in several controls, %Δ[HHb] increase was greater in migraineurs indicating a stronger imbalance between the delivery and utilization of cerebral O_2_.

The present study bears some limitations. Our sample size is small and heterogeneous. Although we found significant differences between the groups, the observations need to be confirmed in larger studies, and also by the use of brain imaging techniques. A confounding effect of cutaneous blood flow on the NIRS-derived cerebral oxygenation seemed to have no effect on cerebral [HHb] as shown in a previous study (26). In addition, it has also been shown that sumatriptan induces both intra- and extracranial vasoconstriction (16). Thus, we argue that the NIRS findings observed in migraineurs do reflect impaired cerebrovascular responses.

To conclude, the observed exaggerated right-side increases in prefrontal NIRS parameters represent disturbances of the tooth clenching-related cerebral (de)oxygenation in individuals with migraine. Mechanisms behind the findings remain unclear but may include impaired stress reactivity together with sensitization of the trigeminovascular system and impaired regulation of the CBF. Although this finding may help to explain the worsening effect of masticatory dysfunctions on migraine, the role of tooth clenching as a migraine trigger calls for further studies involving larger sample sizes.

## Author Contributions

NZ, A-PR, JL, and MN: Conceived and designed the experiments; NZ, JL, A-PR, SR, and AV: Performed the experiments; NZ, JL, and A-PR: Analyzed the data; NZ and A-PR: Wrote the paper; SS, JL, SR, AV, PK, and MN: Revised the paper; PK and MN: Supervised the study.

### Conflict of Interest Statement

The authors declare that the research was conducted in the absence of any commercial or financial relationships that could be construed as a potential conflict of interest.
